# Modeling Neurodegenerative Disorders in *Drosophila melanogaster*

**DOI:** 10.3390/ijms21093055

**Published:** 2020-04-26

**Authors:** Harris Bolus, Kassi Crocker, Grace Boekhoff-Falk, Stanislava Chtarbanova

**Affiliations:** 1 Department of Biological Sciences, University of Alabama, Tuscaloosa, AL 35487, USA; hjbolus@crimson.ua.edu; 2 Genetics Graduate Training Program, School of Medicine and Public Health, University of Wisconsin–Madison, Madison, WI 53705, USA; Klcrocker@wisc.edu; 3 Department of Cell and Regenerative Biology, School of Medicine and Public Health, University of Wisconsin–Madison, Madison, WI 53705, USA

**Keywords:** *Drosophila*, neurodegeneration, neuroregeneration

## Abstract

*Drosophila melanogaster* provides a powerful genetic model system in which to investigate the molecular mechanisms underlying neurodegenerative diseases. In this review, we discuss recent progress in *Drosophila* modeling Alzheimer’s Disease, Parkinson’s Disease, Amyotrophic Lateral Sclerosis (ALS), Huntington’s Disease, Ataxia Telangiectasia, and neurodegeneration related to mitochondrial dysfunction or traumatic brain injury. We close by discussing recent progress using *Drosophila* models of neural regeneration and how these are likely to provide critical insights into future treatments for neurodegenerative disorders.

## 1. Introduction: *Drosophila* as a Model System for Studies of Human Disease

The common fruitfly *Drosophila melanogaster* has been used as a genetic model system for more than 100 years. Because *Drosophila* are inexpensive to maintain and reproduce rapidly, an enormous repertoire of genetic technologies has been created over the past century [1]. *Drosophila* possess extensive homology with humans at the genetic level making them a useful model for investigation of the cellular and molecular processes underlying development and disease [2]. Over the past three decades, a variety of human diseases also have been modeled in *Drosophila*, including many affecting the nervous system [2,3,4]. Human diseases for which there are *Drosophila* models are curated by the Bloomington *Drosophila* Stock Center (https://bdsc.indiana.edu/stocks/hd/index.html). The *Drosophila* studies are facilitated by the fact that the *Drosophila* nervous system is complex and possesses many features of our own nervous system including: eyes, olfactory organs, gustatory organs, auditory organs, a ventral nerve cord (spinal cord analog), peripheral sensory neurons for proprioception and pain, and a brain [5] and the generation of huge collections of mutants that impact neural development [6,7,8,9,10,11,12,13,14,15,16,17]. Moreover, multiple rigorous assays to score neurodegeneration can be used in *Drosophila*, providing reliable measurements for the effects of the disease process. Such assays include examination of eye morphology and retinal structures by light microscopy, vacuolization of the central brain using histological staining, lifespan analysis, locomotor performance measurements using a climbing assay as well as assessment of neuromuscular junction morphology to determine potential synaptic abnormalities [18,19]. Immunohistochemical techniques can be used to label specific subtypes of brain cells such as dopaminergic neurons by using an anti– Tyrosine hydroxylase antibody, or to examine the accumulation of deposits such as amyloid plaques, which are a hallmark of the neuropathology accompanying Alzheimer’s Disease using Thioflavin S labeling [18,20].

**Figure 1:**
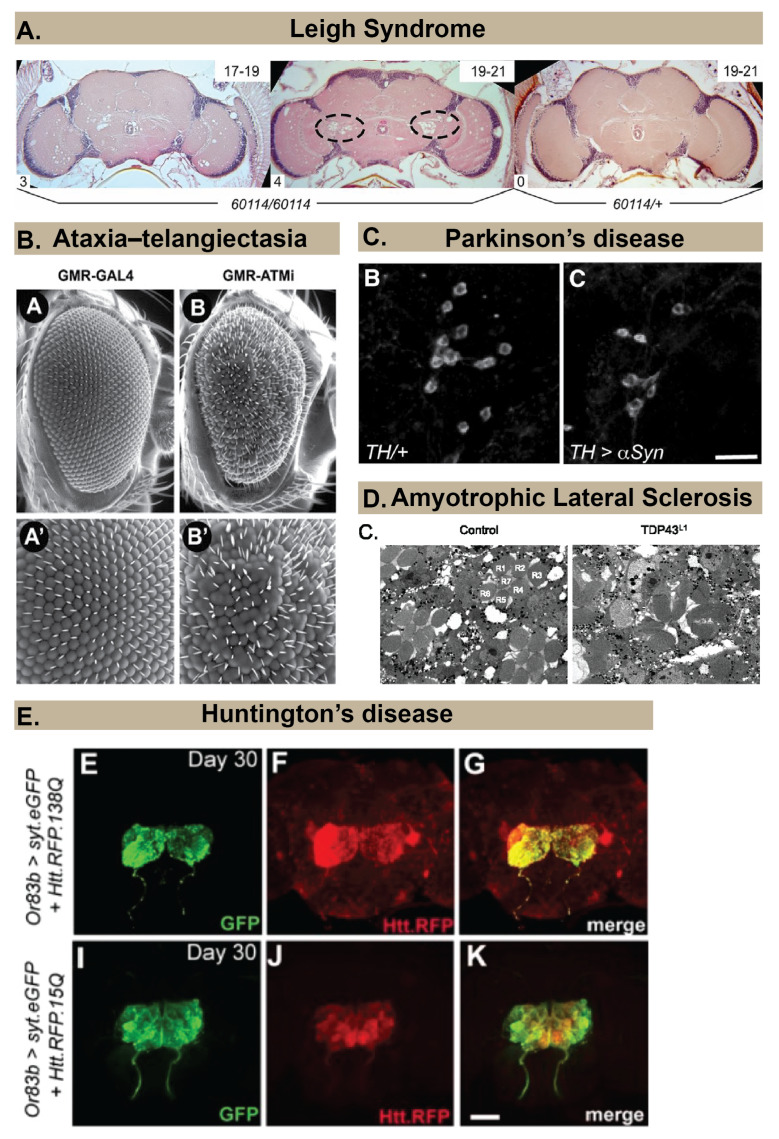
Examples of approaches to examine neuropathology in *Drosophila* models of different human neurodegenerative diseases. (**A**) Spongiform pathology in a *Drosophila* model of Leigh Syndrome, revealed by histology and hematoxylin and eosin (H&E) staining that shows the appearance of holes in the brain neuropil of *60*,*114* mutants (*ND23* mutants) but not in heterozygous controls (*60114/+*). Image copyright and permission to use the image were obtained from [21]. (**B**) Rough eye phenotype (B and B’ for magnified image) observed in a *Drosophila* model of Ataxia Telangiectasia using scanning electron microscopy. Image copyright and permission to use the image were obtained from [22]. (**C**) Loss of dopaminergic neurons in a *Drosophila* model of Parkinson’s Disease is revealed by immunohistochemistry using an anti–Tyrosine Hydroxylase antibody. Image copyright and permission to use the image were obtained from [23]. (**D**) Neurodegeneration in photoreceptors (labeled R1–R7) of ommatidia in a *Drosophila* model of Amyotrophic Lateral Sclerosis (right image) is revealed using Transmission Electron Micrographs. Image copyright and permission to use the image were obtained from [24]. (**E**). Progressive spreading of Red Fluorescent Protein (RFP)-labeled Huntingtin within the brain is revealed by immunohistochemistry in a *Drosophila* model of Huntington’s Disease. Image copyright and permission to use the image were obtained from [25].

In this review, we focus on several major neurodegenerative diseases being modeled in *Drosophila* (Figure 1). The diseases discussed here include adult–onset diseases such as Alzheimer’s Disease, Parkinson’s Disease, Dementia with Lewy Bodies, Amyotrophic Lateral Sclerosis (ALS), Frontotemporal Dementia (FTD) and Huntington’s Disease. We also review existing models of Ataxia Telangiectasia, which is a childhood–onset multiorgan disorder, characterized by progressive neurodegeneration as well as emerging models of neurodegenerative diseases with mutations in mitochondrial genes or the *Drosophila brain tumor* gene. We also discuss Traumatic Brain Injury (TBI) model that is being used to model Chronic Traumatic Encephalopathy (CTE). We conclude with some likely future directions of the *Drosophila* neurodegeneration field that include use of this powerful model to investigate neural regeneration and how these studies may lead to clinically relevant therapeutics.

## 2. Neurodegenerative Diseases Modeled in *Drosophila*

### 2.1. Alzheimer’s Disease

Alzheimer’s Disease (AD) is a form of dementia that usually manifests after the age of 65. It is characterized by memory loss, mood and behavior changes, the presence of specific protein aggregates in the brain, and a reduction in brain regions including the hippocampus and temporal lobes [26,27,28]. AD is one of the leading causes of death in the US; based on data from the 2010 census, 4.7 million individuals over the age of 65 had AD, and an estimated 13.8 million people in the US will have AD by 2050 [29,30]. Although there are no known cures, treatment options include diet and lifestyle interventions, as well as medications that alleviate the symptoms and/or progression of the disease [31,32].

The etiology of AD is the subject of ongoing research, and different hypotheses have been proposed to account for the variety in associated risk factors and physiological changes. The most prominent of these is the amyloid hypothesis, which holds that the buildup of characteristic extracellular amyloid–beta (Aβ) aggregates causes pathology, notably neurodegeneration. Pathogenic, extracellular Aβ42 is produced by sequential cleavage of the intramembrane amyloid precursor protein (APP) (in *Drosophila*, amyloid precursor protein–like (APPL)) by BACE1 (β–site APP cleaving enzyme–1), and γ–secretase. In contrast, non–pathogenic cleavage of APP is carried out by α–secretase [33,34,35]. Another characteristic protein, Tau also is implicated in the amyloid hypothesis. Under normal conditions, Tau binds to microtubules; however, when Tau is hyperphosphorylated, it detaches and forms intracellular aggregates, destabilizing the microtubules and thus decreasing neurotransmission. The cause of Tau hyperphosphorylation is not known, but some data suggest that amyloid pathology contributes, or that they share some mutual causation through a mechanism such as innate immunity. Indeed, activation of the innate immune system and chronic inflammation have been implicated in multiple neurodegenerative disorders [36]. The role of *Drosophila* brain immunity in the contexts of injury and neurodegeneration is reviewed in [37]. Other hypotheses focus on observations related to Tau tangles, cholinergic dysfunction, mitochondrial dysfunction and oxidative stress, calcium homeostasis, vascular dysfunction, glia–mediated inflammation, metal ion toxicity, and poor lymphatic clearance. These proposed mechanisms are all interrelated, often by the role of Aβ aggregates, and all may contribute to the development of AD [35].

Models of AD in *Drosophila* can be divided into those using mutations in the *Drosophila* orthologs of human disease genes, transgenic constructs carrying alleles of human disease–causing genes, and models used to study the effects of environmental stressors on Aβ toxicity (Table 1). Human genes for which *Drosophila* models have been generated include *BACE1*, *BACE2*, *PS1*, *PS2*, *APP*, *MEGF10*, *CD2AP*, *SNRPN*, *PTPRD*, *XYLT1*, *FERMT2*, *CELF1*, *MAST4*, *ITGAM*, and *ITGA9* [38,39,40]. Transgenic constructs have been used to target Aβ production and toxicity; they have also been used to study the role of Tau in the pathology of AD [40,41,42,43,44,45,46,47,48]. Environmental stressors that modulate AD progression and Aβ toxicity include iron, copper, zinc, and light exposure [48,49,50,51,52].

*Drosophila* homologs of AD–associated genes have provided insights into the human genes implicated in the development of AD as well as the pathways that contribute to the disease. The gene Draper (in humans, MEGF10), plays a role in the glial engulfment of Aβ, reducing neurotoxicity in a *Drosophila* model of AD [38]. In another study of 87 *Drosophila* genes, each with a human homolog identified in GWAS as an AD–associated genomic locus, nine were found to strongly affect the toxicity of Tau: *CD2AP* (*cindr*), *SNRPN* (*SmB*), *PTPRD* (*Lar*), *XYLT1* (*oxt*), *FERMT2* (*Fit 1*, *Fit 2*), *CELF1* (*aret*), *MAST4* (*CG6498*), *ITGAM* (*scb*), and *ITGA9* (*scb*) [39]. The proteins encoded by *CD2AP* and *FERMT2* both function with integrins in cell adhesion and signaling; ITGAM and ITGA9 produce α–subunits for integrin receptors; and PTPRD and XYLT1 also function in cell adhesion [39,53,54,55,56].

The human peptide Aβ42 is well known for forming extracellular plaques in AD. Human Aβ42 has been fused with various signal peptides for secretion in transgenic *Drosophila*, targeted by an anti–Aβ42 antibody, and expressed with computer–modeled single amino acid substitutions [41,42,43,44,45,46,47]. Moreover, in humans, the protein encoded by the gene APP carries the Aβ peptide and is cleaved by BACE1 and γ–secretase before secretion. Transgenic constructs in *Drosophila* have investigated APP, BACE1, and pathogenic Psn (the *Drosophila* ortholog of a γ–secretase constituent) separately and in combination [20,33,57,58,59,60,61].

Environmental factors, including diet, lifestyle, and chemical exposure, are known to contribute to AD in humans [62,63]. The metals iron, copper, and zinc; each found in the human diet; have been studied in *Drosophila* models of AD, using a variety of methods to increase and decrease exposure. Copper and zinc supplementation exacerbate Aβ42 toxicity, while leveraging chelators, expression of detoxifying proteins, and expression of transport proteins ameliorate toxicity [50,51]. Iron chelators, when overexpressed, can also attenuate Aβ42 toxicity and when expression of these chelators is reduced, toxicity increases [49,52]. Reflecting a different aspect of lifestyle and environment, a unique study using a Tau model of AD found that disruption of the circadian rhythm with dim light exposure increased neurodegeneration [48].

### 2.2. Lewy Body Dementias: Parkinson’s Disease and Dementia with Lewy Bodies

Lewy Body Dementias (LBDs) are characterized by aggregates of α-synuclein (α–syn) within cells of the brain. These aggregates are called Lewy bodies. There are two types of LBD: Parkinson’s disease (PD) and Dementia with Lewy Bodies (DLB). Overexpression of α–syn in *Drosophila* could be considered to model PD, DLB, or both. However, publications to date refer to α–syn overexpression in *Drosophila* as PD models. We maintain that convention here and therefore do not discuss DLB further. PD is a neurodegenerative disease that affects individuals over the age of 45 at a rate of 572/100,000 in North America. The number of people with PD in the United States is expected to reach 930,000 in 2020 [65]. The characteristic symptoms of PD include tremor and posture instability, which are caused by a loss of midbrain dopaminergic (DA) neurons that deliver dopamine to the basal ganglia [66]. Other brain structures also are affected, such as the cerebral cortex, post–commissural putamen, and olfactory tubercle, leading to diverse symptoms [66]. There are no known cures for the condition, but medications that target dopamine receptors, such as levodopa and dopamine agonists, are effective at treating the symptoms [67]. Other non–pharmacological treatments, such as deep brain stimulation and exercise therapy, may also be effective [68].

Among the molecular mechanisms contributing to PD pathology are neuroinflammation, defects in α–syn proteostasis, mitochondrial dysfunction, oxidative stress, perturbation of calcium homeostasis and defects in axonal transport [67]. Because the etiology of PD is multifactorial, a variety of models have been generated in *Drosophila* to reflect known contributing factors (Table 2). Orthologous genes, transgenic constructs carrying human genes, and environmental factors all have been investigated. The Parkinson’s disease–related genes that have exploitable homologs in *Drosophila* include *PARK2*, *PINK1*, *LRRK2*, *DJ–1*, *UCH–L1*, *HtrA2*, *GBA*, and *Tau* [69,70,71,72,73,74,75,76,77,78,79,80]. α–Syn and Pael–R do not have *Drosophila* homologs, and are studied using transgenic models [79,81]; human transgenes also have been introduced for other genes including LRRK2 and Tau [79,82,83]. Moreover, environmental stressors, such as the widely used pesticides rotenone and paraquat, have been tested on *Drosophila* [84,85,86].

Functions of the *Drosophila* orthologs of genes associated with PD can be investigated using mutant flies or by tissue and/or cell–specific overexpression or knock down using binary expression systems [88]. The *PARK2* gene codes for the protein Parkin, which targets abnormal proteins for degradation. The PD–related proteins Pael–R and α–Syn are among those surveilled by PARK2 [79]. Importantly, *Drosophila* brains, as with human brains, have dopaminergic (DA) neurons. Thus, the consequences of particular mutations and gene overexpression can be examined in DA neurons. In *Drosophila*, PINK1 protein is important for mitochondrial function, and *PINK1* mutants have fewer DA neurons and exhibit both olfactory dysfunction and motor deficits [69,75,76,80]. *Drosophila LRRK2* loss–of–function mutations also reduce DA neuron numbers and cause impaired locomotor activity [71]. DA neuron–specific knockdown of the *Drosophila* ortholog of *UCH–L1*, *dUCH*, leads to Parkinson’s disease–like phenotype illustrated by the loss of DA neurons, while its overexpression leads to caspase–dependent cell death in eye imaginal discs, aberrant patterning of the pupal retina and a rough eye phenotype in the adult [77]. The HtrA2 protein has protease activity and is involved in apoptosis. Knockdown of HtrA2 function in *Drosophila* DA neurons and photoreceptor cells decreases lifespan, motor ability, and ommatidia number [72]. The gene GBA encodes β–glucocerebrosidase, which is necessary for preventing accumulation of glucosylceramides. GBA mutations in *Drosophila* result in DA cell death, motor deficits, and decreased lifespan [73]. The protein Tau can form neurotoxic inclusions that are implicated in both human PD and AD [70]. Overexpression of *Drosophila* Tau in mushroom body neurons results in learning and memory defects [87]. Tau toxicity in *Drosophila* is increased by both over– and under–expression of LRRK2, and is characterized by loss of tyrosine hydroxylase (TH)-immunoreactive neurons [89].

Because flies lack orthologs of human α–Syn or Pael–R, overexpression of human cDNAs has been used to investigate their functions in *Drosophila*. Overexpression of variants of human α–Syn in *Drosophila*, leads to locomotor defects, Lewy body formation in the brain and retinal degeneration [81]. Co–expression of the human proteins α–Syn and Tau, in a *Drosophila* model, leads to the formation of inclusions of ubiquitinylated proteins that disrupt functions of the cytoskeleton, causing neurodegeneration [83]. Overexpression of wild type and mutant human LRRK2 in *Drosophila* leads to degeneration in photoreceptor cells and neurons along with symptoms including motor deficit and decreased lifespan [82].

The pesticides rotenone and paraquat have been linked to the development of PD in humans, and *Drosophila* models of PD have been used to investigate the mechanisms by which this occurs [84,85,86,90]. Rotenone inhibits mitochondrial Complex I, causing oxidative stress. In flies, rotenone causes dose–dependent symptoms including motor deficits and selective DA neuron loss. Moreover, the PD medication L–dopa can be used to treat the motor deficits but not DA neuron loss [84]. Paraquat exposure in *Drosophila* also induces oxidative stress and DA neuron loss [85]. These changes are similar to those observed in post–mortem samples of paraquat–exposed PD patients [91,92]. Recent studies in *Drosophila* have demonstrated that paraquat exposure also leads to deregulated innate immune responses [90]. It remains unclear whether deregulation of the innate immune response is a primary contributor to neurodegeneration following paraquat exposure; however, activation of the innate response has been linked to neurodegeneration in other contexts (e.g., [93]).

### 2.3. Amyotrophic Lateral Sclerosis and Frontotemporal Dementia

ALS is characterized by the progressive degeneration of motor neurons. ALS is a relatively rare, but rapidly progressing, neurodegenerative disease that usually leads to death within 5 years of diagnosis. Familial ALS (FALS) accounts for ~10% of ALS cases, while sporadic ALS (SALS) accounts for the remaining ~90% [94]. A variety of genes have been implicated in ALS. Seven of these genes have been used to generate *Drosophila* ALS models (Table 3). These are: *C9ORF72*, *TDP-43*, *FUS*, *VAPB*, *UBQLN2*, *VCP*, and *SOD–1*. Overexpression, reduced expression, and expression of mutant versions of these genes have been used productively in *Drosophila*. A variety of assays also have been employed including measurement of lifespan, assessment of locomotion; examination of neuromuscular junction (NMJ) phenotypes; quantification of retinal degeneration and sensory neuron dendritic branching.

Frontotemporal dementia (FTD) is a group of disorders characterized by degeneration of the frontal and temporal lobes of the brain. It often has an early onset. Genes that contribute to FTD include: *C9ORF72*, *FUS*, *VCP*, *TDP–43*, *MAPT/tau*, *CHMP2B*, *PGRN*, *TBK1*, and *TMEM106B* (reviewed in [95]), thus there is overlap with other neurodegenerative diseases including: ALS (*C9ORF72*, *FUS*, *VCP* and *TDP–43*), AD (*tau*) and PD (*tau*). *C9ORF72, FUS*, *VCP*, and *TDP–43* studies in *Drosophila* are included in Table 3, while *tau* studies are listed in Table 1 and Table 2.

The most common contributing factor to ALS is a particular repeat expansion in the gene *C9ORF72* that contains hundreds or thousands of the intronic hexanucleotide repeat (G_4_C_2_)_n_ [124,125]. Hexanucleotide repeat expansion (HRE) has been found in more than 5% of SALS patients and 39% of white American and European FALS patients, although it may be less common in other ethnic groups [126]. Repeat RNA can be neurotoxic. However, repeat–associated non–AUG (RAN) translation from these RNAs also can give rise to dipeptide repeat (DPR) proteins, which can be neurotoxic [127,128].

Several strategies have been used in *Drosophila* to introduce precise G_4_C_2_ repeats and to investigate the potential neurotoxicity mechanisms (Table 3). In one study, as few as 30 repeats were found to be sufficient to cause neurodegeneration [105]. A subsequent study compared a variety of RNA–only expression methods, which was accomplished by interspersing stop codons that prevented dipeptide repeat (DPR) protein production. In this study, the RNA carrying the HRE did not result in toxicity, and the DPR proteins encoded by the hexanucleotide repeats were thought to mediate neurotoxicity [104]. Consistent with this, when the effects have been compared of expressing RNA that would code for different dipeptide combinations without using the G_4_C_2_ motif, only arginine–containing DPR proteins were neurotoxic [107]. The *Drosophila* studies contrast with results in zebrafish where both DPR proteins and clusters of the mutant RNA are neurotoxic [129,130].

Both protein and RNA aggregates typically are observed in motor neurons of ALS patients post–mortem. Furthermore, these aggregates commonly contain both ubiquitin and TDP–43, thereby uniting multiple ALS genes in a common, proteostasis–defective, program. TDP–43 encodes the transactive response (TAR) DNA–binding protein, which can bind to both DNA and RNA. Mutations in TDP–43 account for ~4% of FALS cases. TDP–43 protein is normally found in the nucleus, but localizes the cytoplasm in 90% of ALS patient samples. Indeed, cytoplasmic aggregates of TDP–43 are found in ~90% of SALS brain and spinal cord specimens, making these aggregates one of the most reliable ALS diagnostics [94]. TDP–43 is a heterogeneous nuclear ribonucleoprotein (hnRNP) with demonstrated roles in transcription, mRNA splicing, and mRNA transport. It recognizes a UG–repeat sequence in target RNAs. Multiple mechanisms for TDP–43 toxicity have been proposed. One possibility is that TDP–43 functions in part by suppressing the incorporation of cryptic exons into mRNAs; without normal TDP–43 activity, aberrant transcripts are made that encode aberrant proteins. These RNAs and proteins overwhelm the degradation machinery, forming neurotoxic aggregates.

The *Drosophila* ALS models provide unique and powerful tools for understanding the etiology of ALS. Sophisticated genetic analyses that are not feasible in other systems have permitted the identification of both cell–autonomous and non–autonomous mechanisms of neurotoxicity [99] and facilitated analysis of protein vs. RNA contributions to neurotoxicity [104,107]. In addition, application of advanced genetic techniques has permitted the identification of interacting loci for known ALS genes [131,132,133,134,135]. These genetic interactions, in turn, have provided insights into the molecular mechanisms underlying neurodegeneration in ALS patients and provide a platform for the assaying of potential ALS therapeutics [136].

### 2.4. Huntington’s Disease

Huntington’s disease (HD), as with ALS, is caused by repeat expansion mutations. It involves a trinucleotide repeat that results in a polyglutamine (polyQ) segment of 36 or more units in the Huntingtin (Htt) protein [137,138]. It is usually diagnosed between the ages of 30 and 50, and symptoms include progressive problems of coordination, learning ability, decision making, and mood, although it can begin earlier in life with different signs and symptoms [139]. It is most common in European, North American, and Australian populations at 5.7 cases per 100,000 people, as compared to 0.4 cases per 100,000 people in Asian populations [140]. Life expectancy after diagnosis is only 17–20 years and no known treatments can slow the disease’s progression, although some can address symptoms such as chorea [139,140].

Because the *Drosophila* Huntingtin (dHtt) does not have expanded polyQ in its amino terminus [141], most models of HD in *Drosophila* introduce the mutant human gene transgenically, and focus on large polyQ domains as versus studying the entire protein (Table 4). Much of the variation in *Drosophila* models of HD is based on which segments of the Htt protein are expressed. In some studies, expression of fragments of the gene such as exon one only or the first three exons was used, while in others, large segments such as a 12–exon fragment or the sequence encoding the entire protein were used [142,143,144,145]. To introduce compounds of interest, inhibitors of polyQ aggregation such as QBP1 (polyglutamine binding peptide) and bivalent polyQ peptides have been transgenically expressed, genes such *NMNAT* (nicotinamide mononucleotide adenylyltransferase) have been overexpressed, HDAC (histone deacetylase) inhibitors have been administered through food, and polyQ aggregation inhibitors have been delivered using nanoparticles [144,146,147,148]. Of course, alongside overexpression of genes such as *NMNAT*, which leads to reduction in mutant Htt aggregation by promoting autophagic clearance, loss–of–function mutations and conditional expression (after symptoms appear) have also proven useful for investigating pathology and treatment [148]. Treatment with HDAC inhibitors was shown to halt polyglutamine–induced toxicity and improve lethality. Moreover, assays including survival, photoreceptor quantification, circadian rhythmicity, and motor performance are conducive to screening for treatments or deficiency mutations [142,145]. While not a direct assay for neuropathology, circadian rhythm changes are strongly correlated with neurodegeneration in humans as well as animal models [149,150].

### 2.5. Ataxia Telangiectasia (A–T)

Mutations in the human Ataxia Telangiectasia Mutated (*ATM*) gene lead to a variety of pathologies, including increased risk of certain cancers, increased risk of infections, problems with motor control, and neurodegeneration [154]. ATM encodes an atypical protein kinase involved in the repair of double strand DNA breaks. The *Drosophila* homolog of ATM has several names, including dATM and tefu (telomere fusion) and was used to model the disease (Table 5). Similar to human patients, *Drosophila* carrying *dATM/tefu* mutations exhibit neurodegeneration. Furthermore, analysis of dATM/tefu function in *Drosophila* has provided critical insights into the mechanisms underlying neurodegeneration in A–T patients. Specifically, loss of kinase activity in glial cells was shown to lead to activation of the innate immune response and the death of both glia and neurons [155,156]. Furthermore, because activation of the innate immune response has been linked to neurodegeneration in multiple contexts [90,93,157,158], the *Drosophila* studies support the idea of a shared neurodegenerative mechanism underlying multiple disorders, including A–T and AD. More recently, the *Drosophila* model of A–T has been used to screen 2400 compounds for possible A–T therapeutics. These studies identified 10 lead compounds, including one that provided additional insights into the cellular mechanisms underlying A–T pathologies [159].

### 2.6. Mitochondrial Gene Mutations and Neurodegeneration

Mitochondrial dysfunction has long been associated with neurodegenerative diseases (reviewed in [160]). AD, PD, and HD, among others, are all known to be potentiated by defects in mitochondrial dynamics. This is perhaps not surprising when one considers that neurons have extremely high energy requirements [161]. More recently, forward genetic screens in *Drosophila* have been used to identify genes that are so critical to mitochondrial function in neurons that their mutation leads to neurodegeneration, even in the absence of other predisposing mutations (Table 6). We describe two examples here.

In 2017, the Bellen laboratory reported that mutations in Nardilysin (dNRD1) were defective in the folding of the mitochondrial enzyme α–ketoglutarate dehydrogenase, which is a rate–limiting enzyme for the Krebs Cycle [162]. The same group had previously shown that mutations in *dNRD1* led to neurodegeneration in the *Drosophila* retina [163]. Upon further investigation, mutations in *dNRD1* were found to lead to increased levels of α–ketoglutarate which impaired autophagy via an increase in mTORC1 activity [162]. The work was important both because it offered a molecular mechanism for the neurodegeneration observed in the mutants and a potential therapeutic target – mTORC1 – for neurodegenerative disorders caused or enhanced by mutations in *dNRD1*. Indeed, rapamycin was shown to alleviate the neurodegeneration caused by loss of either *dNRD1* or *OGDH* mutations [162], providing a clear proof of principle for the approach. We note, however, that because mTORC1 exerts pleiotropic effects, it may not be an ideal target for neurodegeneration therapeutics.

In 2018, the Ganetzky laboratory reported on the identification of a new allele of the nuclear–encoded mitochondrial Complex I enzyme, *ND23* [21]. *Drosophila ND23* mutations cause progressive neurodegeneration and early death. Another Complex 1 protein, NDUFS8, previously had been implicated in a human disorder called Leigh Syndrome [164]. Leigh Syndrome manifestations include early, progressive neurodegeneration with loss of cognitive and motor function. A confusing aspect of Leigh Syndrome has been the variation in phenotype among patients carrying the identical mutant alleles of NDUFS8. Loewen and Ganetzky now offer both a *Drosophila* model for Leigh Syndrome and an explanation for Leigh Syndrome phenotypic variability. In particular, they found that the mitochondrial genotype modifies the severity of the neurodegeneration in *ND23* mutants and identified a mutation in the mitochondrially encoded ATPase 6 as a strong candidate enhancer of *ND23* mutations [21]. Like ND23 and NDUFS8, ATPase 6 is a component of Complex I. Another Complex I mutation in the *Drosophila* gene *ND75*, homolog of human *NDUFS1*, has been shown to contribute to neurodegeneration [165]. The work underscores the utility of *Drosophila* for understanding neurodegenerative disorders and provides a model for the testing of potential therapeutics.

### 2.7. The Brain Tumor Gene and Neurodegeneration

A novel genetic model for neurodegeneration recently was described in *Drosophila* [166] (Table 7). This model consists of a new mutation in the TRIM–NHL (tripartite motif–NCL–1, HT2A, and LIN–41) protein encoded by the *Drosophila brain tumor* (*brat*) gene. Mutations in human TRIM proteins have been associated with neuropathologies, including AD [167] and axonal neuropathy [168]. However, the new mutation, *brat^cheesehead^* (*brat^ch^*^s^), is unusual in leading to both brain tumors and progressive neurodegeneration. While deletions of *TRIM3* frequently are found in primary human gliomas [169], the simultaneous presence of neurodegeneration and overgrowth is rare and raises the intriguing question of whether one leads to the other. Epidemiological studies have identified positive associations between PD and an increased risk of malignant brain tumors [170,171], while genetic studies have shown that mutations in the human E3–ubiquitin ligase–coding gene *PARK2* are associated with several malignancies in addition to early onset PD [172]. Thus, it seems likely that *brat^chs^* flies may serve as an excellent model for identification of still unknown mechanisms underlying neurodegenerative diseases and for the testing of potential therapeutics.

### 2.8. Drosophila Traumatic Brain Injury and Neurodegeneration

In 2013, the first *Drosophila* model of closed–head TBI was published by the Wassarman and Ganetzky laboratories [173] (Table 8). Similar to humans, TBI in *Drosophila* leads to temporary incapacitation, ataxia, innate immune response activation, neurodegeneration and death [173]. The neurodegeneration is analogous to the CTE observed in human TBI patients. Over the intervening seven years, much has been learned about the factors influencing in TBI outcomes in *Drosophila*. These factors include age and diet as well as genetic background [174,175]. Being able to study the mechanisms underlying neurodegeneration in controlled genetic backgrounds is extremely powerful and already providing insights into both genetic and environmental variables that can contribute to neurodegeneration or to neuroprotection.

The standard TBI protocol in *Drosophila* involves four impacts spaced at 5–minute intervals. A standard outcome measure is the percent of injured flies that die within the first 24 h following the injury. A survey of more than 200 “wild type” *Drosophila* strains derived from a single wild type population [176], revealed that post–TBI mortality is influenced tremendously by genetic background with some strains exhibiting as little as 10% mortality and others exhibiting 60% mortality [175]. Moreover, using mortality as a measure, TBI outcomes were found to be worse in older adults than in younger adults [174].

Notably, restricting food intake after TBI was shown to have beneficial effects, paralleling TBI outcomes in humans, where increased hyperglycemia (e.g., as seen in patients with diabetes) is linked to significantly increased risk of death after TBI [175]. These results suggest that the secondary injuries leading to organismal death are similar in *Drosophila* and humans and that further studies in *Drosophila* are likely to provide additional new information that will help us understand the complex consequences of TBI [175].

Gene expression studies have permitted the identification of genes that are up– or down–regulated following *Drosophila* TBI. The up–regulated genes include components of the *Drosophila* innate immune system [174], some of which previously have been linked to neurodegeneration in *Drosophila* [93,157]. This raises the possibility that pharmacological control of innate immunity programs in human patients could reduce secondary injuries and therefore prevent adverse TBI outcomes.

In recent years, the utility of this model has become sufficiently clear that other laboratories are now using it [177,178,179,180,181,182]. Because of the parallels between *Drosophila* and human responses to TBI, there are multiple future applications for this model. These include evaluating the utility of various drugs in treating TBI in the clinic [183]. Future applications of this work will include testing the efficacy of these same pharmaceutical agents in preventing genetically induced neurodegeneration. In addition, because TBI patients often require surgery, not only for the head injury but also for other injuries sustained in parallel, the *Drosophila* model should also prove useful for the safety of individual anesthetics for TBI patients [184].

## 3. Looking Ahead

In summary, *Drosophila* are a powerful model in which to gain insights into human neurodegenerative disorders. Studies in *Drosophila* have made major contributions to our understanding of neurodegenerative disorders. For instance, the importance of mitochondria to PD was discovered in flies [69,76,80], as was the toxicity of arginine–containing dipeptide repeats in ALS [107]. Candidates from human GWAS can also be readily validated in *Drosophila*, as shown in [39]. The availability of *Drosophila* models for diseases such as AD, PD, and HD provides opportunities for the discovery of molecular mechanisms that affect disease progression and tools for the identification of therapeutics. More recently, *Drosophila* have emerged as a model in which to study neural regeneration. A variety of models have been created, including several in which to investigate axon or dendrite regrowth after injury as well as an adult brain model for the simultaneous analysis of degeneration and regeneration after brain injury (Table 9). An important direction for future research will be to apply what we are learning about neural regeneration to the neurodegenerative disease models to test whether we can slow or reverse specific types of neurodegeneration. For instance, one could imagine using CRISPR/Cas9 to correct a genetic defect in a subset of cells within the brain and then activating those cells to replace neurons and/or glia that were lost or damaged. Using this type of approach, one might first pre–empt Lewy body formation in neural stem cells, then coax those same stem cells to replace lost dopaminergic neurons in a PD model. These types of experiments could be done in *Drosophila*, paving the way for future studies in mammalian models and human patients. Because of the shared developmental genetics of the *Drosophila* and human nervous systems, it seems likely that *Drosophila* will prove as fruitful for modeling neural regeneration as it has for modeling neural degeneration.

## Figures and Tables

**Table 1:** *Drosophila* models of Alzheimer’s Disease.

Alzheimer’s Disease			
Drosophila Model	Developmental Stage	Assay Used for Neuropathology	References
Drosophila orthologs of human genes			
Pan–neuronal and photoreceptor–specific expression of Drosophila dBACE and APPL to produce dAβ	Adult	Toluidine blue histological staining for retinal degeneration, Thioflavin S staining for amyloid deposits, immunohistochemistry using anti–dAβ, fast phototaxis assay, TEM for fibrillary aggregates formation and degeneration	[40]
APPL null mutants	Adult	Histology for brain morphology, fast phototaxis assay, olfactory acuity assay, shock reactivity assay, odor conditioning assay, optomotor assay	[64]
Overexpression of human transgenes			
Pan–neuronal and photoreceptor–specific expression of Aβ40 and Aβ42 fused to rat pre–proenkephalin signal peptide (SP)	Larva, Adult	Larvae: immunostaining and confocal microscopy for Aβ42 accumulation in imaginal eye discs Adult: SEM and light stereomicroscopy for eye morphology, lifespan, immunostaining with anti–Aβ (6E10) for Aβ42 accumulation, toluidine blue histological staining for ommatidial organization	[41]
Pan–neuronal expression of Aβ40, Aβ42 and Aβ42^arc^ fused to Drosophila Necrotic protein SP	Adult	Lifespan, climbing assay, immunostaining with anti–Aβ (4G8) for Aβ42 accumulation, SEM for eye morphology	[42]
Photoreceptor–specific and mushroom body–specific expression of Aβ42 fused to Drosophila Argos SP	Adult	Light microscopy and SEM for retina structure, light microscopic histology of frontal eye sections for vacuolar degeneration, immunostaining and Thioflavin S staining for Aβ42 accumulation in eyes	[43]
Photoreceptor–specific expression of Aβ42 and blocking	Larva, Pupa, Adult	3^rd^ Instar Larvae: immunostaining for eye imaginal disc development and Aβ42 accumulation, TUNEL staining for eye imaginal disc cell death, Pupae: immunostaining for eye development and Aβ42 accumulation Adult: immunostaining for eye development and Aβ42 accumulation, histology for photoreceptor morphology, SEM for eye morphology,	[44]
Expression of various mutated Aβ42 peptides for the effect of specific amino acid substitutions on toxicity	Adult	Lifespan, locomotor assay, immunohistochemistry using anti– Aβ42, Thioflavin T staining for rates of Aβ42 aggregation, TEM for Aβ42 aggregate morphology	[45]
Expression of various mutated Aβ42 peptides for the effect of specific amino acid substitutions on toxicity	Adult	Lifespan	[46]
Pan–neuronal and muscle–specific expression of Aβ42, exposure to exogenous Aβ42, and treatment with anti–Aβ42 antibody (6E10)	Larva	3rd Instar Larvae: Electrophysiology for synaptic transmission, FM1–43 dye imaging for neurotransmitter release, Thioflavin S staining for amyloid deposits	[47]
Pan–neuronal and photoreceptor–specific expression of two human Tau variants, manipulation of light exposure	Adult	Lifespan, histology, and light microscopy to quantify neurodegeneration, climbing assay, immunohistochemistry for pTau accumulation, light microscopy for eye morphology	[48]
Pan–neuronal expression of human APP and BACE1 separately and in combination, treatment with a γ–secretase inhibitor	Adult	Lifespan, fluorescence microscopy for defects in the whole–brain and mushroom body structure, immunostaining with anti– Aβ (6E10), Thioflavin S, and X–34 for amyloid deposition, climbing assay, conditioned courtship suppression assay	[57]
Expression of human BACE1 and late–onset induction of human APP	Adult	Lifespan, climbing assay, immunostaining with anti–Aβ (6E10) for amyloid deposition, fluorescence microscopy for defects in the whole–brain and mushroom body structure, conditioned courtship suppression assay	[58]
Combined models of Drosophila orthologs and overexpression of human transgenes			
Knockdown of orthologs of human CD2AP, SNRPN, PTPRD, XYLT1, FERMT2, CELF1, ITGAM, ITGA9, MAST4 in Drosophila overexpressing human Tau^V337M^	Adult	Light microscopy for eye morphology	[39]
Expression of Drosophila Psn, Drosophila APPL, human APP, and human BACE, separately and in combination	Adult	Histological staining for retinal degeneration, Thioflavin S and immunostaining with anti– Aβ (4G8) for Aβ accumulation in retinas, survival assay, lifespan	[20]
Aβ42^arc^ overexpression, Draper inhibition, overexpression of Draper/MEGF10	Adult	Lifespan, Thioflavin S and anti–Aβ (6E10) immunostaining for Aβ, climbing assay, histological sectioning for vacuole quantification	[38]
Photoreceptor–specific expression of human Aβ42 in eyes, supplementation with zinc or copper, treatment with chelators, and overexpression of MTF–1	Larva, Adult	Larva: relative eclosion rate Adults: Stereomicroscopy for ommatidia structure, climbing assay	[50]
Pan–neuronal expression of Aβ42, treatment with an iron chelator, and RNAi knockdown of ferritin	Embryo, Adult	Embryos: Hatching efficiency assay Adults: Survival assay, Thioflavin T staining for amyloid aggregation	[49]
Photoreceptor–specific Aβ42 expression, over– and under–expression of an immunophilin, mutation in a copper transporter, and treatment with an exogenous copper chelator	Adult	Lifespan, light microscopy for eye morphology	[51]

**Table 2:** *Drosophila* models of Parkinson’s disease.

Parkinson’s Disease			
Drosophila Model	Developmental Stage	Assay Used for Neuropathology	References
Drosophila orthologs of human genes			
Parkin mutants	Adult	TH immunostaining, climbing assay	[78]
PINK1 mutants PINK1 knock down in DA neurons	Adult	Lifespan, TH immunostaining, chemotaxis assay, dopamine enzyme immunoassay, HPLC for DA tissue and dopamine levels, fast–scan cyclic voltammetry, RT–PCR for DAT and GAPDH2, Western blot for TH, mobility assay	[69,75,76,80]
LRRK2 mutants	Adult	Climbing assay, TH immunostaining	[71]
DJ–1 mutants exposed to hydrogen peroxide, paraquat and rotenone	Adult	Lifespan, TH immunostaining	[74]
Photoreceptor cell–specific overexpression of dUCH and DA neurons–specific knockdown of dUCH	Larva, Pupa, Adult	SEM for eye morphology, activated–Caspase 3 immunostaining, TH immunostaining, larval crawling assay, adult climbing assay, pupal retinal mispatterning determination	[77]
HtrA2 knockdown in DA neurons and photoreceptor cells	Adult	Lifespan, climbing assay, SEM for eye morphology	[72]
Double heterozygous GBA mutants (CG31414 and CG31148)	Adult	Lifespan, TH immunostaining, climbing assay	[73]
Overexpression of dTau in mushroom body neurons	Adult	Survival up to 30 days of age, learning and memory assays	[87]
Overexpression of human transgenes			
Co–expression of Tau and Alpha–Synuclein (α–syn)	Larva, Adult	Activated–caspase 3 immunostaining, larval NMJ morphology, TH immunostaining, SEM for adult eye morphology	[83]
Pan–neuronal, photoreceptor cell– and DA neurons–specific overexpression of wild type, A30P and A53T α–syn	Adult	H&E staining, TH immunostaining, Lewy body detection using Ubiquitin immunostaining, TEM for neuronal α–syn inclusions, climbing assay, Toluidine blue staining of tangential retinal sections	[81]
Pan–neuronal, photoreceptor cell– and DA neurons–specific overexpression of LRRK2 and LRRK2–G2019S–2	Adult	Lifespan, climbing assay, TEM for photoreceptor morphology in ommatidia, TH immunostaining, actometer test	[82]
Overexpression of Pael–R in DA neurons	Adult	TH immunostaining	[79]
Toxin exposure			
Rotenone	Adult	TH immunostaining, climbing assay	[84]
Paraquat	Adult	TH immunostaining, lifespan, climbing assay, jumping assay, Dopamine levels	[85,86]

**Table 3:** *Drosophila* models of Amyotrophic Lateral Sclerosis.

Amyotrophic Lateral Sclerosis			
Drosophila Model	Developmental Stage	Assay Used for Neuropathology	References
Drosophila Orthologs of Human Genes			
FUS (Cabeza in Drosophila)			
Ectopic expression of wildtype and disease–mutated FUS	Larva, Adult	Immunostaining to detect altered subcellular localization of Cabeza in larval motor neurons, adult eye morphology, lifespan	[96]
VCP			
siRNA knockdown	Drosophila cell culture	Western blotting to detect accumulation of high molecular weight forms of ubiquitin	[97]

VAPB (Vap33 in Drosophila)			
Ectopic expression of mutant and wild type VAP–33	Larva, Adult, Drosophila cell culture	Larvae: Larval wing imaginal disc, larval NMJ, adult brain, adult muscle, analysis of mitochondrial morphology in flight muscle; analysis of endoplasmic reticulum (ER) fragmentation in larval brains, Adult: analysis of eye morphology, analysis of cell death, ubiquitinated aggregates Drosophila cell culture: and ER stress in cultured Drosophila S2 cells	[98,99,100,101,102,103]
Overexpression of Human Transgenes			
C9ORF72			
Pan–neuronal expression of RNA–only constructs expressing (G_4_C_2_)_106_ repeats with both intronic (nucleus) and polyadenylated (cytoplasm) sense and antisense transcripts Pan–neuronal expression of UAS–RNA sense polyA constructs expressing 800–1000 and >1000 (G_4_C_2_) repeats	Adult	Lifespan, negative geotaxis, light microscopy for eye morphology	[104]
Eye and pan–neuronal expression of UAS–(G_4_C_2_)_3_ and UAS–( G_4_C_2_)_30_ constructs in eye and motor neurons	Adult	Lifespan, light, and SE microscopy for eye structure and ommatidia loss, locomotion assay	[105]
UAS–(G_4_C_2_)_48_ expression in Class IV epidermal sensory dendritic arborization neurons	Larva	Dendritic branching analysis using confocal microscopy	[106]
Eye and pan–neuronal expression of UAS constructs containing 3, 36 and 103 pure, and 36, 108 and ~288 RNA–only (G_4_C_2_) repeats	Embryo, Adult	Stereomicroscopy for eye structure, lifespan, egg–to–adult viability	[107]
Ectopic expression of UAS constructs containing 8, 28 and 58 (G_4_C_2_) repeats	Larva, Adult	Larval locomotion, larval salivary gland nuclear envelope morphology, adult eye morphology	[107,108,109,110]
Ectopic expression of UAS constructs containing 36 protein–coding and 160 RNA–only (G_4_C_2_) repeats	Larva, Adult	Dendritic branching, lifespan, eye morphology	[110]
Ectopic expression of UAS constructs containing 30 (G_4_C_2_) repeats	Cultured Drosophila S2 cells, larval salivary gland, adult eye	Nuclear import, adult eye morphology	[111]
TDP–43			
Reduced and ectopic expression of wild type TDP–43	Larva, Adult	Larval and adult locomotion, larval NMJ morphology, adult mushroom body morphology, adult learning	[112]
Ectopic expression of wild type and disease–mutated variants	Larva, Adult, cultured motorneurons	Larval NMJ morphology, larval motorneuron death, larval glia, adult sleep	[113,114]
Ectopic expression of wild type and disease–mutated variants	Larval eye imaginal discs, Adult	Subcellular localization, lifespan, locomotor activity	[113]
Ectopic expression of wild type and mutant TDP–43 with and without a chaperone protein	Larva, Adult	Larval protein aggregation, adult eye morphology,	[115]
FUS			
Ectopic expression of wildtype and disease–mutated FUS	Larva, Adult	Subcellular localization in larval motor neurons, adult eye morphology, lifespan	[96]
Ectopic expression of wildtype and disease–mutated FUS	Adult	Adult eye morphology,	[116,117,118]
Motor neuron expression of wildtype and disease–mutated FUS	Larva, Adult	Larval brain size, larval motorneuron subcellular localization, larval locomotion, adult eye morphology	[119]
UBQLN1/2			
Ectopic expression of wild type and disease variants	Adult	Measurement of TDP–43 levels in adult eye lysates	[120]
Co–expression of human UBQLN and TDP–43	Larva, Adult	Larval NMJ morphology, lifespan, measurement of TDP–43 levels in adult head lysates, adult eye morphology, adult locomotion assays	[24,121]
VAPB			
Expression of wild type human VAPB in Drosophila neurons	Larva	Larvae: Rescues lethality, NMJ morphology, and NMJ electrophysiology of loss–of–function mutations in Drosophila VAP–33	[122]
SOD–1			
Ectopic expression of wild type and disease variants	Adult	Lifespan, locomotion, number of motorneurons, neuronal SOD–1 accumulation, glial stress response	[123]

**Table 4:** *Drosophila* models of Huntington’s disease.

Drosophila Model	Developmental Stage	Assay Used for Neuropathology	References
Huntington’s Disease			
Drosophila orthologs of human genes			
Fly dHtt does not express polyQ in its N–terminus	N/A	N/A	[141]
Overexpression of human transgenes			
Transgenic expression of a Q48 peptide or Htt Exon1p in neurons	Adult	Lifespan, photoreceptor morphology count	[144]
Transgenic expression of various Q48 constructs	Adult	Locomotor, photoreceptor morphology count	[151]
Transgenic expression of Q108 and Q48 peptides, transgenic expression of bivalent polyQ peptides	Adult	Lifespan, photoreceptor morphology count	[152]
Expression of Q127 and Q20 peptides	Adult	SEM and light microscopy for retina morphology, light microscopy for pigmentation defects, staining with FITC for presence of polyQ aggregates	[153]
Expression of mRFP–tagged N–terminal fragments of human Q15 or Q138 peptides	Adult	Lifespan, locomotion, activated–Caspase 3 immunostaining, immunostaining for brain size	[148]
Expression of an mRFP–tagged N–terminal fragment of human Q15 or Q138 peptides containing exons 1–12	Adult	Immunofluorescence for spreading of Huntingtin aggregates in the brain	[25]
Expression of several 3– or 1–exon sections of mutant Htt with various polyQ lengths in clock neurons and ventral lateral neurons (sLNvs), RNAi knockdown of heat shock protein (Hsp)	Adult	Htt–eGFP fusions to track and quantify aggregation, sLNv count, rhythmicity, confocal imaging for PER protein intensity, transcript levels of Hop–associated proteins	[145]
Expression of Q93 and Q20 peptides	Adult, Larva	Adult: locomotion Larva: crawling assay	[146]
Temperature–inducible expression of a Q15 or Q138 12–exon fragment of the human Htt gene, or expression of a 548 amino acid Q0 or Q128 segment of human Htt	Adult, pharate adult, larva	Adult: RFP tag for imaging of Htt aggregation and localization Pharate adult: lethality Larvae: viability past 2nd instar for small molecule screen, Fluorescence recovery after photobleaching for aggregate growth	[142]
Expression of full–length Q128 or Q16 human Htt	Adult, larva	Adults: Western blot for Huntingtin levels, photoreceptor morphology count, locomotion, flying assay, confocal microscopy to count neuronal projections into IFMs Larvae: immunohistochemistry for third–instar larval NMJ count, EJP amplitudes, Ca^2+^ imaging	[143]

**Table 5:** *Drosophila* models of Ataxia Telangiectasia.

Ataxia Telangiectasia			
Drosophila Model	Developmental Stage	Assay Used for Neuropathology	References
Drosophila orthologs of human genes			
ATM^8^ mutants and knockdown of ATM	Adult	Climbing assay, lifespan, histological staining for vacuole quantification, immunostaining with anti–Casp^Act^ for prevalence of apoptosis	[155]
ATM^8^ mutants and knockdown of ATM	Adult	Concurrent climbing assay, lifespan, histological staining for vacuole quantification, immunostaining with anti–Casp^Act^ for prevalence of apoptosis	[156]
ATM^3^, ATM^4^, and ATM^8^ mutants	Adult	Percent eclosion, lifespan	[159]

**Table 6:** *Drosophila* models of neurodegenerative mitochondrial gene mutations.

Mitochondrial Gene Mutations and Neurodegeneration			
Drosophila Model	Developmental Stage	Assay Used for Neuropathology	References
Drosophila orthologs of human genes			
ND23 mutants	Adult	Climbing assay, bang–sensitivity assay, lifespan, histological staining for vacuole quantification	[21]
dNRD1 mutants, OGDH mutants, and knockdown	Adult	Electroretinogram recordings for neuronal function, histology for retinal morphology	[162]
ND75 knockdown	Adult	Lifespan, climbing assay, histological staining for vacuole quantification, immunostaining for cleaved PARP to quantify caspase activity	[165]

**Table 7:** *Drosophila* model of a neurodegenerative brain tumor.

Brain Tumor			
Drosophila Model	Developmental Stage	Assay Used for Neuropathology	References
Drosophila orthologs of human genes			
brat^chs^ mutant, brat^chs^; pcna–GFP and brat^chs^; CG15864^MB04166^ double mutants	Adult	Histological staining for vacuole quantification, climbing assay, immunostaining with anti–cleaved Dcp–1 for prevalence of apoptosis	[166]

**Table 8:** *Drosophila* models of neurodegenerative traumatic brain injury.

Traumatic Brain injury			
Drosophila Model	Developmental Stage	Assay Used for Neuropathology	References
Injury from the High–Impact Trauma device	Adult	Lifespan, histological staining for vacuole quantification	[173]
Stab injury to the brain through the right eye	Adult	Lifespan, climbing assay, mobility assay	[183]

**Table 9:** *Drosophila* models of neuroregeneration.

Neuroregeneration					
Drosophila Model	Developmental Stage	Injured Tissue	Assay Used for Neuropathology	Assay Used for Neuroregeneration	References
Nerve crush injury	Larva	Motor and sensory neuron axons	Visualization of degenerating distal stump using GFP reporters	Visualization of regenerating proximal stump using GFP reporters	[185,186,187]
In vivo laser axotomy	Larva	Sensory neuron axons	Visualization of degenerating distal stump using GFP reporters	Visualization of axon regrowth using GFP reporters	[185,188,189,190]
In vivo laser dendriotomy	Larva	Sensory neuron dendrites	n.d.	Visualization of dendrite regrowth using GFP reporters	[189,190,191]
In vitro axotomy	Larva	Motor neuron axons	n.d.	Visualization of axon regrowth using GFP reporters	[192]
In vivo axon pruning and remodeling	Pupa	Mushroom body of the brain axon pruning and remodeling	Immunostaining of fixed samples	Immunostaining of fixed samples	[193,194,195,196,197]
Ex vivo axon pruning and remodeling	Pupa	Mushroom body of the brain axon pruning and remodeling	Immunostaining of fixed samples	Immunostaining of fixed samples	[198]
Ex vivo axotomy	Adult	Brain sLN–v neurons	Visualization of degenerating distal stump using GFP reporters	Visualization of axon regrowth using GFP reporters	[199]
Olfactory neuron axotomy	Adult	Antennal olfactory neuron axons	Visualization of degenerating distal stump using GFP reporters	n.d.	[196,200,201]
In vivo axotomy	Adult	Wing sensory neuron axons	Visualization of degenerating distal stump using GFP reporters	Visualization of axon regrowth using GFP reporters	[202,203,204]
Traumatic Brain Injury (TBI)	Adult	Various brain regions	histology	n.d.	[173,183]
Penetrating Traumatic Brain Injury (PTBI)	Adult	Various brain regions	TUNEL assays,	Cell proliferation, lineage tracing	[183,205,206,207]

## Definitions and Abbreviations

Aβ	Amyloid–beta
AD	Alzheimer’s Disease
ALS	Amyotrophic Lateral Sclerosis
APP	Amyloid Precursor Protein
APPL	Amyloid–beta–like protein
A–T	Ataxia Telangiectasia
BACE	Beta–secretase
Cas9	CRISPR Associated Protein 9
CD2AP	CD2-Associated Protein
CELF1	CUGBP Elav-Like Family member 1
CRISPR	Clustered Regularly Interspaced Short Palindromic Repeats
CTE	Chronic Traumatic Encephalopathy
C9ORF72	Chromosome 9 Open Reading Frame 72
DA neurons	Dopaminergic neurons
DAT	Dopamine transporter
DCP–1	Death caspase–1
DPR	Dipeptide repeat expansion
eGFP	Enhanced green fluorescent protein
EJP	Excitatory junction potential
FERMT2	Fermitin family homolog 2
FITC	Fluorescein isothiocyanate
FM1–43	Fluorescent dye used for the real–time measurement of exocytosis and endocytosis in living cells
FUS	Fused in Sarcoma
GBA	Glucosylceramidase beta
GFP	Green fluorescent protein
GWAS	Genome–Wide Association Study
H&E	Hematoxylin and Eosin
HD	Huntington’s disease
HPLC	High–performance liquid chromatography
IFM	Indirect flight muscle
ITGAM	Integrin alpha M
ITGA9	Integrin alpha 9
LRRK 2	Leucine-rich repeat kinase 2
MAST4	Microtubule-associated serine/threonine kinase family member 4
MEGF10	Multiple EGF-like-domains 10
mTORC1	Mammalian target of rapamycin Complex 1
NMJ	Neuromuscular junction
Ommatidia	Clusters of photoreceptors and supporting cells that compose the adult eye
PARP	Poly(ADP–ribose) polymerase
PD	Parkinson’s disease
PER	Period
PINK1	PTEN-induced kinase 1
pTAU	Phosphorylated Tau
pTBI	Penetrating Traumatic Brain Injury
PTPRD	Receptor-type protein tyrosine phosphatase delta
RFP	Red Fluorescent Protein
RNA	Ribonucleic acid
RT–PCR	Reverse transcription PCR
SEM	Scanning Electron Microscopy
siRNA	Small interfering RNA
SNRPRN	Small nuclear ribonucleoprotein polypeptide N
SOD	Superoxide dismutase
TBI	Traumatic Brain Injury
TEM	Transmission Electron Microscopy
TH	Tyrosine hydroxylase
TRIM-NHL	Tripartite motif-NCL-1/HT2A/LIN-41
TUNEL	Terminal deoxynucleotidyl transferase dUTP nick end labeling
UAS	Upstream activating sequence
UBQLN2	Ubiquilin 2
UCH-L1	Ubiquitin carboxy-terminal hydrolase L1
VAPB	Vesicle-associated membrane protein-associated protein B/C
VCP	Valosin-containing protein
XYLT1	Xylosyltransferase 1
X–34	Fluorescent dye used to stain for amyloid depositions
